# Simulation and Computer Study of Structures and Physical Properties of Hydroxyapatite with Various Defects

**DOI:** 10.3390/nano11102752

**Published:** 2021-10-17

**Authors:** Vladimir Bystrov, Ekaterina Paramonova, Leon Avakyan, José Coutinho, Natalia Bulina

**Affiliations:** 1 Institute of Mathematical Problems of Biology, The Branch of Keldysh Institute of Applied Mathematics, RAS, 142290 Pushchino, Russia; ekatp@yandex.ru; 2 Physics Faculty, Southern Federal University, 344090 Rostov-on-Don, Russia; laavakyan@sfedu.ru; 3 Department of Physics & I3N, University of Aveiro, Campus Santiago, 3810-193 Aveiro, Portugal; jose.coutinho@ua.pt; 4 Institute of Solid State Chemistry and Mechanochemistry, Siberian Branch of RAS, 630128 Novosibirsk, Russia; bulina@solid.nsc.ru

**Keywords:** hydroxyapatite, modeling, density functional theory, defects, vacancies, substitutions, structural and optical properties, band gap, electronic density of states, nanomaterials

## Abstract

Simulation and computer studies of the structural and physical properties of hydroxyapatite (HAP) with different defects are presented in this review. HAP is a well-known material that is actively used in various fields of medicine, nanotechnology, and photocatalytic processes. However, all HAP samples have various defects and are still insufficiently studied. First of all, oxygen and OH group vacancies are important defects in HAP, which significantly affect its properties. The properties of HAP are also influenced by various substitutions of atoms in the HAP crystal lattice. The results of calculations by modern density functional theory methods of HAP structures with these different defects, primarily with oxygen and hydroxyl vacancies are analyzed in this review. The results obtained show that during the structural optimization of HAP with various defects, both the parameters of the crystallographic cells of the HAP change and the entire band structure of the HAP changes (changes in the band gap). This affects the electronic, optical, and elastic properties of HAP. The review considers the results of modeling and calculation of HAP containing various defects, the applied calculation methods, and the features of the effect of these defects on the properties of HAP, which is important for many practical applications.

## 1. Introduction

Hydroxyapatite (HAP) is a mineral compound from the apatite group, the basic salt of calcium and phosphoric acid (calcium phosphate) with the formula Ca_5_ (OH) (PO_4_)_3_, a widely employed multifunctional material in biomedicine, health care, biology, ecology, catalysis, and for environmental remediation [1,2,3,4,5,6,7,8,9,10]. First of all, HAP is the main mineral component of mammalian hard tissues (bone and teeth). Along with the organic component (collagen) and living bone cells (osteoclasts, osteoblasts, and osteocytes), HAP crystallizes within the gaps of stacked tropocollagen fibrils, forming and strengthening the bone structure [1,2,3,4,9]. Due to innate bioactivity and biocompatibility, HAP is a widely used material in medicine for bone and dental surgery. Here, hydroxyapatite is used as a filler to replace parts of lost bone (in traumatology, orthopedics, hand surgery) and as a coating for implants to promote new bone growth.

Since the mechanical properties of HAP differ from bone material, implants are made from the most suitable material (usually titanium is used), but its surface is covered with HAP [3], which provides better adhesion and reproduction of bone cells on its surface [2,4]. It is important to note here that biological HAP (bio-HAP) found in living organisms differs from an ideal synthetic mineral crystal of HAP in stoichiometric imbalance and various defects [5]. It is the presence of such defects that creates inhomogeneities in the structure of HAP, leading to local changes in the electrical potential and topography of the HAP surface, which ensures successful survivability of bone and growth of bone tissue.

Therefore, it is so important to study and understand the role of defects in HAP: what defects arise here and how they affect the properties of HAP. Then, we will be able to purposefully synthesize HAP with the necessary defects that provide the desired properties.

Besides medical applications, HAP can be used in environmental remediation for heavy metals absorption and removal, especially for bivalent cations such as Pb (II), Cd (II), and Zn (II) [6]. Another feature exhibited by HAP is the capability of degrading hazardous organic and inorganic chemicals, both in air and water, upon exposure to ultraviolet (UV) light, demonstrating the photocatalytic activity [6]. 

HAP also exhibits a strong affinity for organic compounds and living organisms, which is very useful, for instance, for waste water treatment and for environmental remediation, and even in cancer treatment [4,9]. Again, defects and dopants play a central role here, which requires their investigation. One effective way to study defects in HAP is precisely computer simulation and computational studies.

Recent studies of the structure and various properties of hydroxyapatite (HAP) have convincingly shown that actual samples (natural and synthetic), especially of biological origin (from the bone tissues of mammals, fish, and mollusks), are quite different from the ideal crystallographic HAP models, which are usually used in modeling and calculations [1,2,3,4,5,6]. 

It turned out that these actual HAP structures with defects promote better biocompatibility, adhesion, attachment of the bone cells, and their proliferation, since they are closer to the biological HAP, which has its own structural heterogeneities [1,2,3,4]. It is important to establish which HAP properties with defects are most essential here.

HAP samples with defects close to biological ones have not only “incorrect” stoichiometry (their composition, as a rule, differs from the stoichiometric Ca/P ratio ~1.67 [9,10,11,12]), but also have a number of additional numerous defects (oxygen vacancies and whole OH groups, impurities, interstitials, and substitutions of a number of atoms in the crystallographic lattice) [13,14,15,16,17,18,19,20,21,22]. Moreover, the distribution of all these defects in the sample volume is uneven, stochastic, and depends on many conditions of its fabrication: temperature, pressure, heating and cooling rates, the composition of the environment, humidity, etc. [19]. It is clear that any theoretical models and high-precision calculations using a variety of modern methods reflect only part of these complex heterogeneous HAP structures. Nevertheless, by developing these models and calculation methods, and carrying out more and more detailed precise calculations, we can determinate the peculiarities of different studied structural defects and correlate their properties with the set of the experimentally observed data [4,5,6,7,8,9].

It is known that the basic structure of HAP has such specific features as extended structural channels formed by chains of the OH groups (OH-channels) [5,6,7,8,9,13,16]. This feature leads to the possibility of proton movement along these channels [16,17] and the appearance in HAP of the defects such as vacancies of protons, oxygen, and whole OH groups [2,4,5,7,9,14,15,16,17,18,19,20]. Various defects have different effects on the change in the properties of a regular HAP periodic lattice. So, for example, the HAP samples during fabrication and treatment are heated to certain temperatures (about ~500–700 °C or about ~773–973 K [16,17,18,19,20]), and the OH groups leave the samples quite easily. As a result, HAP is dehydrated [7,19,20]. During subsequent cooling, OH groups from the environment are reintroduced into these channels. However, their concentration changes (depending on the humidity of the environment, etc.) and a partial concentration of the OH vacancies will remain in the HAP samples, in some its lattice unit cells, which significantly affects all their properties [2,4,19,20].

It is known that the basic structure of HAP has such specific features as extended structural channels formed by chains of the OH groups (OH-channels) [5,6,7,8,9,13,16]. This feature leads to the possibility of proton movement along these channels [16,17] and the appearance in HAP of the defects such as vacancies of protons, oxygen, and whole OH groups [2,4,5,7,9,14,15,16,17,18,19,20]. Various defects have different effects on the change in the properties of a regular HAP periodic lattice. So, for example, the HAP samples during fabrication and treatment are heated to certain temperatures (about ~500–700 °C or about ~773–973 K [16,17,18,19,20]), and the OH groups leave the samples quite easily. As a result, HAP is dehydrated [7,19,20]. During subsequent cooling, OH groups from the environment are reintroduced into these channels. However, their concentration changes (depending on the humidity of the environment, etc.) and a partial concentration of the OH vacancies will remain in the HAP samples, in some its lattice unit cells, which significantly affects all their properties [2,4,19,20].

Another important and specific defect in HAP are oxygen vacancies [2,4,5,6,8,9,21,22]. They can arise from both the OH group and the PO_4_ group. Moreover, it could be of different types depending on the position of the oxygen atom and the symmetry of the given atomic PO_4_ group [8]. Their formation is possible at the higher temperatures (of the order of ~1100–1300 °C or about ~1373–1573 K) [19,20] or also under radiation exposure [23].

Further important defects are interstitials (in particular, the insertion of hydrogen atoms or protons into HAP lattice structure [2,4,5]) and substitution of some atoms in the HAP structure by others atoms in the HAP unit cell lattice (for example, Fe/Ca, Sr/Ca [5,24,25,26,27,28,29]) and some others (such as Mg/Ca, Se/Ca, Si/P, etc.) [5]. It leads to many important practical applications, because it changes the HAP surface electrical potential [2,4] that initiates higher adhesion and proliferation of various osteo-cells on HAP surface, and this improves the biocompatibility of the implant [2,3,4,5,28]. 

Basically, the main physical and chemical properties of HAP are determined precisely by the presence of various structural defects, such as oxygen and hydroxyl vacancies, interstitials and substitutions of ions, atoms in the structure of HAP [1,2,3,4]. Various modern experimental and theoretical methods are used to study them. Among them, computer modeling and calculations, including first principles ab initio and density functional theory (DFT) methods [5,6,7,8,9,30,31,32,33,34,35,36,37,38,39,40,41,42,43,44,45,46,47,48,49,50,51] are the most important. 

In previous research, using some modern DFT methods (including several types of the hybrid functionals and the theory of many-particle perturbations) and developed software tools (AIMPRO, VASP, and Quantum Espresso (QE) [7,8,38,39,40,41,42,43,44,45,46,47,48,49,50,51,52]) in the modeling and computer studies of the properties of HAP structures with these various types of the defects, it was found that the forbidden band gap Eg of an ideal (defect-free) stoichiometric HAP lattice turns out to be much larger than the data of measurements of the optical absorption characteristics of the usual HAP samples [2,4,18,19]. These calculations show, that Eg can reach values of the order of ~7.4–7.9 eV [7,35]. This significantly exceeds the experimentally observed values for the HAP samples used in practice (Eg ~3.8–4.5 eV) [2,4,9,20]. These recent computational studies of the HAP structure with defects have convincingly shown that the deviations of the calculated band gap Eg of the ideal stoichiometric HAP crystal from the experimentally observed Eg values are caused precisely by the presence of the various defects in the HAP structure [2,4,5,6,7,8,9,36,37].

The calculations of oxygen vacancies (from PO_4_ and OH groups with different symmetry) and total OH-group vacancies in the HAP structure were carried out recently, showing very interesting results [5,6,7,8,9].

These oxygen and OH vacancies were investigated both in the local charge density approximation (LDA) of the DFT [5,6,38,42,43] and in the DFT generalized gradient approximation (GGA) [48,49,50], including the use of the modern types of hybrid DFT functionals and the theory of many-particle perturbations [7,8,39,41,44,45,46,47,48,49,50,51,52,53,54]. These studies of the structural and electronic properties of oxygen vacancies presented in the plane wave formalism [46,47,51] were carried out both in the single unit cell model (in the LDA DFT [5,6]) and in the model of large HAP supercell model (2 × 2 × 2 = 8 unit cells) [7,8,36,37] and (in the GGA DFT [48,49,50]) by using hybrid DFT functionals.

These calculations and studies have shown that, under equilibrium conditions, oxygen vacancies arise not only in the form of a simple vacant oxygen site (in a neutral charge state) [8], but can also be in the form of extended structures occupying several crystal fragments or chemical units (especially in a double plus charge state) [8,36,37]. In this case, the connection between the presence of defects and changes in the optical properties of HAP and the level of its photoelectronic work function is clearly traced [2,4,5]. In fact, here was shown, that for any different approaches and methods of DFT calculations, it is oxygen vacancies that play their decisive role here, changing the optical absorption width Eg of an ideal stoichiometic HAP crystal from its large characteristic values to a lower level of the order of ~3.6–4.2 eV, determined by these introducing HAP defects (oxygen vacancies), which is as usually recorded on HAP experimental samples.

In general, these studies are still far from complete and require further deep continuation.

In this review, we present some recent data on the study of defects, primarily oxygen vacancies (of the various types) and the OH vacancies, obtained by modern methods of computer simulation and first principle of quantum-mechanical calculations based on DFT. In addition, in this work, the calculations are compared with the available experimental data, and an analysis of this comparison is given.

## 2. Computational Details, Main Models, and Methods

### 2.1. Main Methods and Used Software

The calculations of pristine stoichiometric and defective HAP structures and its properties were carried out from the first principles using DFT by AIMPRO code in Local Density Approximation (LDA) [5,6,38,42,43] and by VASP [7,8,39,44,45,46,47,48] in Generalized Gradient Approximation (GGA) within Kohn-Sham formalism [45,46,47] according to the Perdew, Burke, Ernzerhof (PBE) approach [49] or one of the following hybrid density functionals: the HSE of Heyd, Scuseria, and Ernzerhof [54]; the Becke three-parameter (B3LYP) functional [49,50]; the PBE0 functional [55]. These are called *semi-local and hybrid density functional calculations* [7]. 

The LDA method also involves a pseudopotential approach for calculations of each atomic potential and wave function [42,43]. Standard valence configurations were used for this purpose in [5,6]: hydrogen (1s^1^), phosphorus (3s^2^3p^3^), and oxygen (2s^2^2p^4^). For calcium atoms, two different valence configurations were used [5]: (1) with a two-electron (4s^2^) shell, and with a ten-electron (3s^2^3p^6^4s^2^) shell. The charge density and potential terms were Fourier-transformed using plane waves, with a cut-off energy of *E*cut = 300 a.u. [5]. 

For the GGA approach, the projector augmented-wave (PAW) method [51] was used [7,8] to account for [Ca]:1s^2^2s^2^2p^6^3s^2^, [P]:1s^2^2s^2^2p^6^, and [O]:1s^2^ core electrons. Valence electrons were described using plane waves with kinetic energy up to *E*cut = 400 eV [7,8]. For presentation, some structural details and properties calculations of the transformation of DFT files into HyperChem workspace using OpenBabel software [56] as well as the combination with semi-empirical quantum-chemical (QM) PM3 method from HypemChem 8.0 package [40] were used. More computational details are all presented in works [5,6,7,8] and are described below. Some recent calculations were made also using Quantum ESPRESSO [41].

### 2.2. Main Details of HAP Crystal Structure

Hydroxyapatite (HAP) have the general chemical formula Ca_5_(PO_4_)_3_OH [10]. HAP solidifies in the form of an ionic molecular crystal, either with hexagonal (P6_3_/m) or monoclinic (P2_1_/b) symmetry, depending on conditions, and has unit cells enclosing two or four formula units, respectively [10,11,12,13]. The crystal structure and unit cell parameters of HAp with both P6_3_/m and P2_1_/b symmetries, and its atomic positions, were determined using X-ray diffraction [11] (Table 1).

HAP usually crystallizes in a hexagonal crystal system (space group P6_3_/m) under normal conditions, according to temperature and stoichiometry, but can also exist as a monoclinic structure (space group P2_1_/b) [1,2,3,4,5,9,10,11,12,13]. As noted by Elliot [10], direct synthesis of monoclinic phase usually requires very strict adherence to the correct stoichiometry, with a Ca:P ratio of 1.67 [4,9,10,11,12,13].

One of the common structural peculiarities of HAP structure is connected with the pseudo-one-dimensional character of the apatite structure. Са and OH**^-^** ions form a long chain along the main structural *c* axis [9,10,11,12,13,14,15,16,17,18,19], often being named as an ‘OH-channel’ in HAP. For the P6_3_/m phase consisting from two chemical formula units and the HAP general formula is [Ca_10_ (PO_4_)_6_(OH)_2_], where the hydroxyl units OH shows stochastic orientation along OH channels.

Thus, according to X-ray data, this actually makes the material mirror-symmetric along the main axis, which runs along the OH channel. Conversely, when all OH blocks show the same alignment along the hexagonal axis, the mirror plane is lost and the space group symmetry drops to P6_3_. The ordering of the dipoles in the OH-channels, which interact with the chains of the OH-channel, also affects the property of the monoclinic phase. If OH^–^ ions are oriented in parallel, HAP has non-centrosymmetric ordered structure (space group P2_1_), which could reveal piezoelectric properties [17,18,36,37].

The structure of HAP, used to study the effect of defects on its properties, is primarily based on its initial pristine stoichiometric structural phase–hexagonal P6_3_, with a unit cell consisting of 44 atoms and containing structural OH channels with two hydroxyl OH groups in each unit cell [5,6,7,8,9,13,14,15,16,17,18,19,20]. Depending on the orientation of these OH groups, the cells can have different symmetry groups (Table 1): P6_3_/m—for the hexagonal disordered phase (when the orientation of the OH groups is random) and P6_3_—for the hexagonal ordered phase (when the orientation of the OH groups is parallel and directed in the same direction, which creates its own internal polarization, similar to ferroelectrics (see below, for example, in [36,37]).

**Figure 1:**
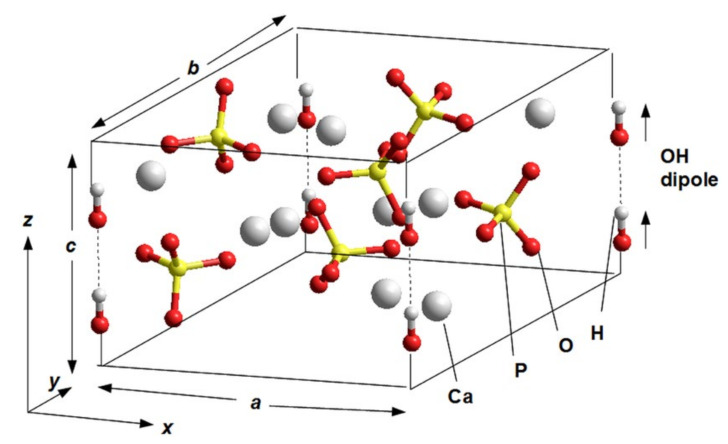
One unit cell model of the Hydroxyapatite (HAP: Ca_10_(PO_4_)_6_(OH)_2_)– in P6_3_ hexagonal phase. All OH groups are oriented in the same direction. They are positioned at the four corners of the unit cell, but only one pair in one corner belongs to this unit cell, the other three pairs belonging to neighboring unit cells (e.g., one OH per unit cell). It also shows the axes of the Cartesian coordinates *x, y, z* and unit cell parameters ***a, b, c***. (Adapted with permission from ref. [5]; IOP Publishing, 2015).

Some earlier calculations were performed using one unit cell model of hexagonal HAP P6_3_ [5,6] (see Figure 1). The main peculiarity of the next study is the introduction of the supercells model made up of 2 × 2 × 2 = 8 HAP unit cells (space group P6_3_) for hexagonal HAP phase both for pristine and defective HAP with OH and oxygen vacancies [7,8] (see below in Section 2.4). 

### 2.3. HAP One Unit Cell Model

The stoichiometric HAP unit cell model for pure (defect-free) lattice in P6_3_ hexagonal phase is presented on Figure 1 [5]. This model was used for the calculations firstly for LDA method [5,6] and then for GGA approach too in several cases [7]. It was used as an initial unit cell model of hexagonal HAP (P6_3_) lattice and for the cases with several of its defects, such as, OH-vacancy, and various O-vacancies.

This one HAP unit cell consists of 44 atoms and contains two OH groups in each periodical unit cell. It should be noted that only one OH-channel with two OH groups is included in the unit cell, while the remaining three OH-channels (with six OH groups) belong to other periodically repeating unit cells.

For monoclinic HAP nanostructures, the unit cell consists of 88 atoms, because the lattice unit cell is doubled in the monoclinic case along the ***b*** axis. Besides this basic hexagonal unit cell of 44 atoms, the supercells totaling 176 atoms, made with two and four unit cells along the ***a*** and ***c*** directions of the hexagonal lattice were constructed to investigate defects in the work [5].

For the unit cell with 44 atoms, we used a 6 × 6 × 8 grid of **k** points (288 points), following the recipe proposed by Mokhorst and Pack [57], while for the large supercells, the grid used was 2 × 2 × 4. All **k**-point sets were folded according to the symmetry of the problem. All atom positions were allowed to relax along the forces acting upon them, until the total energy converged below 10^−5^ eV. After convergence test calculations, a 2 × 2 × 4 **k**-point mesh was found good enough to sample the Brillouin zones (BZs) of both hexagonal and monoclinic cells.

**Figure 2:**
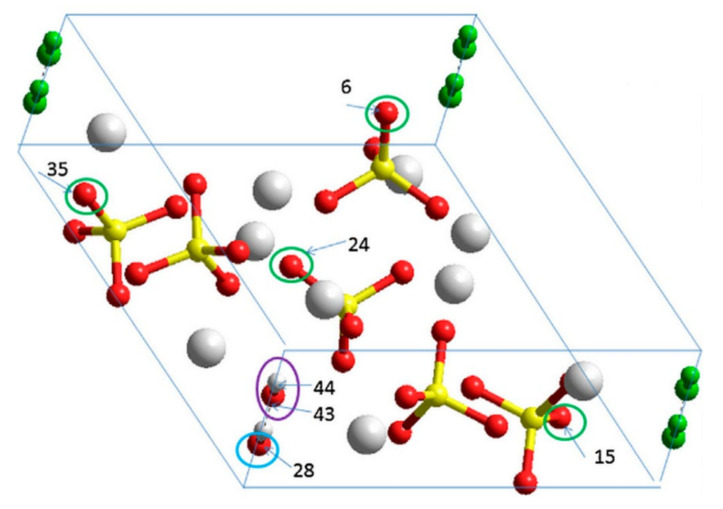
Models and scheme of the atomic positions selected and deleted for modelling presentation of various vacancy-defects in the HAP hexagonal unit cell with initial 44 atoms. Indicated in the colored circles are: (1) in blue—oxygen atom (number 28) from an OH group for creation of an O-vacancy in the OH-channel; (2) in purple—OH group full (with atom numbers 43 and 44) for creation of a complete OH-vacancy; (3) in green—oxygen atoms (with numbers 6, 15, 24, 35) from various differently positioned PO_4_ groups corresponding to an O-vacancy in PO_4_ groups. The green atoms on the other three corners indicate other OH groups, which belong to neighboring periodical repeated unit cells. (Adapted with permission from ref. [6]; Elsevier, 2016).

**Figure 3:**
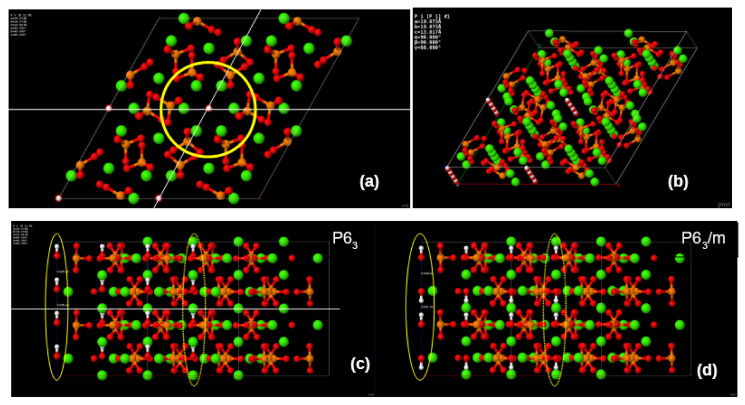
Images of supercell models with 2 × 2 × 2 = 8 unit cell of HAP (visualization using Jmol after DFT calculations: (**a**) top view—along the c axis of the OH channel (a yellow circle highlights the main central part around one OH channel); (**b**) supercell model in isometric projection; (**c**) HAP supercell model in lateral projection for the P6_3_ phase (OH groups are parallel oriented (ordered) in OH channel along c axis and therefore can create a summarily polar state); (**d**) HAP supercell model in lateral projection for the P6_3_/m phase (OH groups are anti-parallel oriented (disordered) in OH channel along c axis and therefore can create a summarily non-polar state).

The HAP unit cell model, containing several defects in the atomic positions of all the vacancies used in this work, is shown in Figure 2. Here, we indicate OH-vacancy and various types of O-vacancies in the HAP unit cell, as deviations from the initial stoichiometric hexagonal HAP structure [6]: (1) an O-vacancy in the OH group of an OH-channel structure (blue circle in Figure 2); (2) a full OH-vacancy from this OH-channel structure (purple circle in Figure 2); (3) various cases of O-vacancies from different positions in PO_4_ groups (green circles in Figure 2).

For GGA approach, the Brillouin zone (BZ) was sampled using a Γ-centered 2 × 2 × 3 mesh of **k**-points. The Hartree-Fock exact exchange was evaluated at the same **k**-point grid used for the DFT potential and stored on a real-space grid of 128, 128, 96 points along **a**1, **a**2, and **a**3 lattice vectors, corresponding ***a, b, c*** (see Figure 1). The experimental lattice constants are ***a***1 **= *a***2 **= *a*** = 9.417 Å and ***a***3 **= *c*** = 6.875 Å [11], corresponding to a grid density of about 14 points/Å along all three directions.

In all these calculations—except for the elastic constant calculations (bulk modulus B) and the DOS calculations—full relaxation of the cell, including changes in volume and adjusting the atomic positions and cell shape (total optimization of structure) was allowed. When calculating the elastic constants, only the relaxation of ionic positions within the strained cells was allowed. In several calculations, e.g., when defects as substitutions of atoms (Ca on Sr, Fe, Mg etc.) were explored, the calculations for fixed former atomic positions were also used, and then their relaxation to the new ones. The optimized data obtained for each HAp structure were taken (files with optimized and fixed coordinates for all unit cell atoms in atomic and Cartesian units were used) and transformed further to another, special HyperChem (*.hin) format (using the Babel program [56]). Then, all these atomic coordinate data files were uploaded into HyperChem software tools for further molecular modeling (with visual options for all studied HAp structures), calculations, and exploration of its physical properties using HyperChem package [40].

### 2.4. HAP Supercell Model

The main feature of our more detailed study is the introduction of the super-cells model made up of 2 × 2 × 2 = 8 polar HAP unit cells (with space group P6_3_) for hexagonal HAP ordered phase (both for initial pristine stoichiometric and defective HAP with oxygen vacancies and with full OH group vacancy) [7,8].

It should be noted that this phase demonstrates polar ferroelectric state with the total polarization along c-axis, due to OH groups oriented parallel and along the OH-channels. While the disordered phase of hexagonal HAP (with space group P6_3_/m) forms non-polar paraelectric state, due to compensation of an opposite anti-parallel orientation of OH groups, which are along these OH channels [36,37].

Oxygen vacancies were introduced in hexagonal supercells made up of 2 × 2 × 2 = 8 polar HAP unit cells (space group P6_3_), comprising a total of 352 atoms (Figure 3). Although the monoclinic phase P2_1_/b was found to be more stable [12,33], they only differ by a few tens of meV per unit cell, with their electronic structures being essentially identical [34].

For the HAP unit cell, we have shown previously that convergence of the electron density and energy is obtained when sampling the band structure with a Γ-centered 2 × 2 × 3 mesh of k-points within the first Brillouin zone. By doubling the size of the cell along all principal directions, reciprocal lattice vectors are contracted by a factor of two so that a 1 × 1 × 2 k-point grid would actually improve on the sampling quality. From convergence tests, we found that the total energy of the 352-atom bulk supercell obtained with a 1 × 1 × 1 grid (Γ-point sampling) differs by less than 0.1 eV from a 1 × 1 × 2-sampled calculation. More importantly, relative energies and ionization energies of defects differ by about 1 meV only. Therefore, all defect calculations employed a Γ-point sampling [7,8].

A full relaxation of such large supercells using plane wave hybrid-DFT is prohibitively expensive. Instead, in works [7,8], defect structures were first found by relaxing all atomic coordinates within PBE, until the maximum force became less than 10 meV/Å. The resulting structures were employed on a second step, where the total energy was obtained within hybrid-DFT by means of a single-point calculation. This procedure was necessary due to the sheer size of the Hamiltonian at hand combined with the use of a plane-wave method. As it was recently shown in [7,52], the relative energies obtained within this methodology are usually affected by error bars of the order of 10 meV or lower.

## 3. Main Results and Discussions

### 3.1. Main Structural and Mechanical Properties of HAP

We start by analyzing the structural and mechanical properties of HAP as initial stoichiometric as well as HAP with considering defects: OH-vacancy and various O-vacancy types. Table 2 and Table 3 compare the calculated structural data (unit cell lattice parameters and volume) and bulk modulus (for some cases) with the respective experimental data. Table 2 presents the data of pristine stoichiometric HAP (computed by various methods and from different experimental data) and data of HAP with one full OH-vacancy (per one HAP unit cell). Table 3 consists of calculated data for various types of O-vacancies and it is shown below.

The results obtained with LDA method (in earlier calculations using AIMPRO [5,6]) and with GGA approximation on the various DFT exchange-correlation functionals: PBE, HSE, B3LYP, and PBE0 (using VASP calculations [7,8]) are presented in Table 2 and Table 3. The calculated lattice parameters for initial pristine stoichiometric HAP lattice within the PBE level approach are in good agreement with the various previously known PBE results (see [48,49,50,51] and references therein).

***Comment 1 for OH groups orientation difference.*** Recently computed data have shown, first, that HAP can coexist at different stages, both hexagonal and monoclinic [5,36,37], as well as in ordered and disordered hexagonal due to the orientation of the OH groups in one unit cell by aligning the OH units in opposite directions: OH-OH is the ordered P6_3_ phase and OH-HO is the disordered P6_3_/m phase. The energy difference between the two phases is DE = E (P6_3_/m)—E (P6_3_) [5,7,8]. This difference, obtained from our various calculations and presented here in Table 2, is: ΔE = ~0.43 eV for PBE (GGA) and ΔE = ~0.132 eV for LDA (AIMPRO) [5]. In a similar PBE calculation in [34], this ΔE ~0.4 eV, and in the most developed model of a large supercell [7,8] is ΔE = 0.39 eV, and the last two energies were obtained from calculations of the completely relaxed B3LYP level [23].

Consequently, during heating (temperature above ~1000 K) and cooling, many OH groups can change their orientation in different unit cells, which ultimately can lead to some stochastic redistribution of them, and the sample will contain a mixture of both types P6_3_ and P6_3_/m hexagonal phase of HAP. It is very difficult to distinguish them experimentally. Therefore, the comparison of the results of our calculations and experimental data must be carried out very carefully, taking into account the possible errors arising also due to this factor of different random orientations of OH groups in experimental samples.

***Comment 2 for two-steps calculation for supercell models.*** For supercell models, structural optimization was made using PBE(GGA) only for initial stoichiometric HAP and after that the calculation in B3LYP was done in the fixed atomic positions in single point (SP) mode. All further calculations of the HAP with defects were performed using PBE(GGA) optimization only for atomic/ionic positions keeping the lattice parameters fixed as it was founded (established) for initial stoichiometric HAP lattice.

The results obtained show the usual ~1% overestimation in relation to the experimental data. This is known to be mostly due to an artificial over-delocalization of the electronic density when the GGA approach is employed. It is shown that the calculations using B3LYP results in improvement of ***c*** lattice parameter, but parameter ***a*** is still overestimated by ~1% [7]. This confirms previous reports that B3LYP generally overestimates the experimental lattice parameters as well [35]. On the other hand, our result differs from the previous B3LYP calculations of HAP, where ***a*** was underestimated by ~1% [35]. More discussion about the reasons of these discrepancy was given in [7].

Table 2 also indicates that the lattice parameters calculated within hybrid DFT are generally closer to the experiments than those obtained using the semi-local functional. The best results are obtained for PBE0 with a deviation of <0.6% in relation to the experiments [7].

It should be noted, that the results obtained earlier in the LDA approximation are the closest to the experimental data on the lattice parameter ***a*** (even slightly better than in the case of a PBE0 with deviation less than 0.6%), while the value of the parameter ***c*** turned out to be more overestimated here (the deviation is almost 1.8%).

The bulk modulus (B) were obtained here usually by fitting the Birch-Murnaghan equation of state [5,61,62,63,64]. The calculated bulk modulus show reasonable agreement with the experiments (see data in 2nd column of Table 2, Refs. [61,62]). The errors (shown in the table) were obtained from the standard fitting procedure. B3LYP calculations show deviation from the measurements by about 3% only, while other methods underestimate the experimental value of B by 7–8%. This level of accuracy is in line with typical discrepancies found for many other insulating materials [63]. It is interesting again to note, that LDA approximation shows here the same value of B as on PBE (GGA) level.

Experimental data on stoichiometric natural and synthetic samples should be used and analyzed rather carefully, since there may be impurity ions (carbonate, nitrate) that affect the lattice parameters. Therefore, the composition of stoichiometric hydroxyapatite should be confirmed by other analytical methods (for example, IR data), in addition to diffraction methods, which is usually used to determine the lattice parameters. Also, synthetic stoichiometric HAP should be well annealed at high temperatures in humid air. Table 2 provides experimental data that mostly meet these requirements [11,58,59,60,61,62]. Below in Section 3.3.1, we discuss this data in comparison with our calculations.

The results of more detailed calculations for the presence of the various types of oxygen vacancies in HAP are presented in Table 3. Here, a complete optimization of the HAP structure (cell parameters and ion relaxation) was carried out only by methods LDA (AIMPRO) and PBE (GGA) in the one unit cell model, since such an optimization has not yet been performed for the supercell model.

#### 3.1.1. Defects (OH-Vacancy and Various O-Vacancies) Influence on Structural and Mechanical HAP Properties

Let us now consider the effect of defects (oxygen vacancies and OH group vacancy) in the HAP structure, which changed in their structural and mechanical properties, and which turns out to be important for many practical and technological applications.

(1) ***Influence of OH-vacancy.*** The data calculated by LDA (AIMPRO) show that the relative changes in the lattice parameters of HAP after the appearance of an OH vacancy (in one HAP unit cell) both increase by small values: δ***a***~0.15% and δ***c***~0.05% (see Table 2).

At the same time, calculations using PBE (GGA) show that the value of the lattice parameter also relatively increases by δ***a***~0.6%, and the parameter c increases by δ***c***~0.5% (see Table 2). For comparison with experimental data, we use here the results of experiments on heating and cooling HAP samples, which should lead to the escape of some OH groups from the OH HAP channel and the formation of OH vacancies in HAP ([58,59,60] in Table 2).

In situ diffractometric studies carried out in the group of Dr. N.V. Bulina showed that the cooling of apatite sample in helium (He) atmosphere, heated to a temperature of 1100 °C, leads to a decrease in parameter ***a*** and an increase in parameter ***c***. Table 2 shows these obtained experimental data. In such conditions, OH vacancies formed at high temperatures remain up to room temperature and the cooled sample is now the oxyhydroxyapatite (OHAP) containing vacancies of OH groups. The changes obtained in the lattice parameters in the presence of OH vacancies in comparison with those known for pristine stoichiometric HAP turned out to be as follows: the relative changes were the largest for the parameter ***a***, the decrease was up to δ***a***~−0.016%, and for the parameter ***c***, an increase by about δ***c****~*0.5%.

If we compare with the initial experimental data [11], then the changes are similar. Thus, these obtained data are very close to the calculated data in the main trend of changes in the lattice parameters during the formation of an OH vacancy: a slight decrease in the lattice parameter ***a*** (and in the calculations there is an increase) and a noticeable increase in ***c*** value for all cases due to the formation of an OH vacancy in the HAP sample. As for the volumes of the cells, with both methods of calculation, a relative increase in volume was obtained (Table 2). For Bulina’s experimental data, the volume slightly decreased. However, if we compare it with the initial volume of [11], then it also increased.

It is important that the calculation results show a similar character of the cell parameter measurement. This means that the chosen model and calculation method are correct and allows us to investigate in more detail the physical reasons for this phenomenon [19].

The calculated values of the modulus B (performed after the LDA method optimized calculation) showed its decrease in this case of the presence of the OH vacancy in HAP sample, which means a decrease in the mechanical strength of HAP samples (Table 2).

It seems that the presence of any vacancy type, including the OH group, should increase the lattice parameters, which is what we get. However, it is likely that in the experiment, out of two vacancies of OH groups, one disappears, and it is occupied by the O^2–^ ion (that is, the hydrogen atom H also disappears here and one oxygen ion remains—this is already forming now the oxyapatite (OAP or OA)). This is possible and leads to the following observed changes in the lattice parameters: HAP (a = 9.4236 Å, c = 6.8802 Å) goes into OHAP (a = 9.4155 Å, c = 6.8835 Å). Moreover, some recent experimental data show that if the OAP is formed here, then the lattice parameters should be as follows: OAP (a = 9.4057 Å, c = 6.8938 Å).

Unfortunately, we have not yet performed calculations of the exact OAP models, containing two OH vacancies per one HAP unit cell. It would be very interesting and useful, and we will definitely do it in the near future. In this case, we suppose to reach decreases of both lattice parameters as experimentally observed by Bulina.

(2) ***Influence of only one O-vacancy from OH group*.** In this case (see in Table 3), the calculated values of the HAP unit cell parameters give some relative decrease in the parameter ***a*** (both for the LDA (AIMPRO) and GGA (PBE/VASP) methods), while the ***c*** parameter also decreases when calculated by the LDA method, but slightly increases when calculated by the GGA (PBE) method. Unfortunately, we have no experimental data for this case. 

(3) ***Influence of the different O-vacancy from PO4 group***. In this case (presented in Table 3), the calculated values of the unit cell parameters were considered for different positions of oxygen atoms that create O-vacancies in various place in the HAP unit cell (for example, see Figure 2). For the model of one unit cell, these positions were selected and calculated by the LDA method in the works [5,6], and in Table 3 these data are given. Similarly, the calculation using GGA (PBE) method was performed and presented here too.

These O-vacancies were computationally investigated in more detail by the GGA (PBE) and B3LYP methods in the work [8] in the HAP 2 × 2 × 2 supercell model. We will analyze these data separately in Section 3.3 below, devoted to supercell model (especially devoted to its electronic and optical properties).

For the cases of O-vacancies from the PO4 group indicated in Table 3, the results obtained by both LDA and GGA (PBE) methods in comparison with initial stoichiometric HAP (hexagonal P6_3_) from Table 2 show that both the unit cell parameters *a* and *c* decrease (as well the unit cell volume), despite some of their differences for the different positions of these selected oxygen atoms, create these O-vacancies. We do not have experimental data for structural analysis in this case, but these cases demonstrate very interesting and important results of their electronic and optical properties, which will be analyzed below.

### 3.2. Electronic and Optical Properties

#### 3.2.1. Electronic Properties of Pure HAP by Various Methods

To study the electronic and optical properties of HAP, both the initial defect-free and those containing various defects, the densities of states of electrons (DOS) at the different energy levels were calculated. This makes it possible to determine the effect of various defects on the energy band structure of these HAP properties.

As a result of LDA calculations (using AIMPRO software [38]) and GGA calculations (using VASP with functional PBE [39,48]), the distributions of the DOS corresponding to the filling of energy levels in the band structure with electrons and the energies of HAP according to the band theory were obtained [5,6,7,8,9,36,37]. This makes it possible to determine the main energy band structure’s parameters—the position of the top of the valence band Ev, the bottom of the conduction band Ec, and the value of the forbidden band gap Eg = Ec − Ev, as well as the position of additional energy levels Ei induced by defects in the internal range of the Eg.

The question now is how defects change this entire band energy structure of the DOS and how close these changes it will be to the experimental values, when creating certain defects in the HAP structure. For these calculations at the first stage [5,6], the defects simulated were created by vacancies into one hexagonal unit cell of HAP consisting of 44 atoms (Figure 1). Figure 2 schematically shows examples of simulated HAP structures with defects such as oxygen vacancies from the OH and PO_4_ groups, as well as a vacancy of the entire OH group. For more details and precise calculations with various DFT hybrid functional we use supercell model consisting of 352 atoms (eight unit cells) (Figure 3) [7,8].

**Figure 4:**
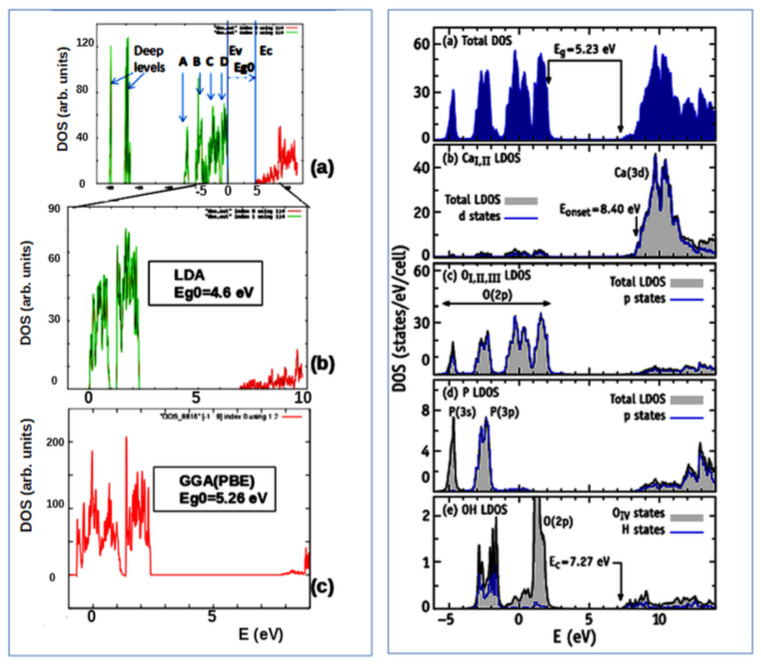
**Left figures:** Density of electronic states (DOS) for HAP unit cell: (**a**) Initial perfect stoichiometric HAP with 44 atoms in hexagonal P6_3_ unit cell lattice, including deep levels and A,B,C,D peaks in valence band computed in LDA by AIMPRO; (**b**) the same with main energies around Eg; (**c**) the same computed in GGA (PBE) by VASP; (Data presented here obtained after calculations using AIMPRO (LDA) [38] and using VASP (PBE-GGA) [39]); **Right figures:** Total density of states of bulk HAP (**a**) and local densities of states projected on calcium (**b**), oxygen in PO4 units (**c**), phosphorous (**d**), and oxygen and hydrogen in OH units (**e**). In (**b**–**d**), the contribution to the LDOS with the dominant angular momentum is represented as a thick line. The results were obtained using the PBE exchange-correlation functional. (Adapted with permission from [7]; AIP Publishing, 2018).

Let us first consider HAP without any defect in the initial pristine stoichiometric hexagonal phase P6_3_ symmetry. Figure 4 (Left figures: a,b,c) shows examples of DOS for perfect stoichiometric defect-free HAP (taking into account both deeper energy levels in the valence band and in the vicinity of the forbidden band) obtained in LDA calculations [5,6]. Similar GGA (PBE) calculations were carried out using VASP software [39]. Figure 4c shows results of DOS from GGA (with PBE functional). Figure 4a–c denotes Eg_0_ = Eg because it refers to a defect-free HAP (see details also Section 3.2.3 below).

Note that the calculations using the LDA and GGA (PBE) approximations generally do not fundamentally differ here, the difference is only in the energies—GGA (PBE) gives a larger value of the band gap Eg~5.26 ± 0.05 eV (for perfect stoichiometric HAP) compared to calculations by the LDA method, which give band gap value Eg ~ 4.6 ± 0.05 eV. These data are close to results of other authors [30,31,32,33,34,35].

Further, Figure 4 (Right figures: (a−e)) shows the total DOS of a HAP unit cell obtained within PBE to be more precise with detailed calculations [7,8], including also local density of states (LDOS) [7]. The shadow plots on subsequent figures [right Figure 4b−e] depict the LDOS projected on several atomic species.

Here, we can see that the calculations using both GGA (PBE) approach do not much differ between one another, the difference is only small in the energies; first GGA (PBE) gives a value of the band gap Eg ~ 5.26 ± 0.05 eV, while second PBE gives a value Eg = 5.23 eV, that is in the accuracy frame and in line with calculations of other authors by this method [9,30,31,32,33,34,35,36,37].

Along with the LDOS of right Figure 4b−d, we plot a thick line representing the dominant angular-momentum component for the corresponding species. In right Figure 4e, we distinguish states projected on O_IV_ and H atoms that form OH molecules. At first glance, Figure 4b,c suggest that the upper end of the valence band is mostly made of O(2p) states, while the conduction band bottom is mostly made of Ca(3d) states. From Figure 4c,d, we find phosphorous 3s-3p states mixing with oxygen 2s-2p states between 5 and 2 eV and they are far below the band gap region. All these results are in line with reports of other authors [9,18,19,30,31,32,33,34,35,36,37].

#### 3.2.2. Electronic Band Structure of Perfect Stoichiometric HAP

Further calculations of HAP perfect stoichiometric structure and properties were developed and performed in [7] using various DFT hybrid functionals. These results obtained demonstrate rise of Eg for perfect stoichiometric HAP using a more developed functional and method. We discuss this point below.

**Figure 5:**
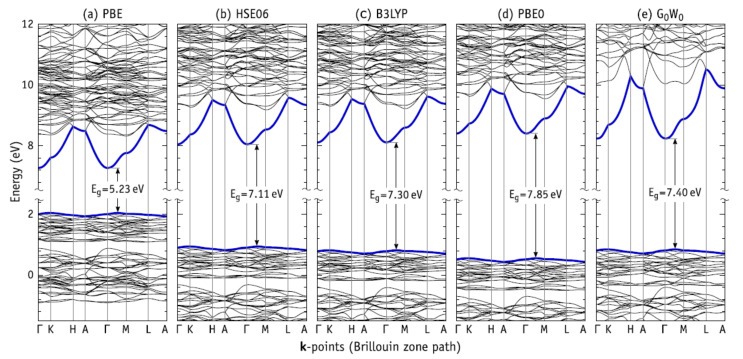
Electronic band structure of bulk perfect stoichiometric hydroxyapatite along a path with breaks at k-points of high symmetry. The calculations were carried out using DFT using functionals: (**a**) PBE—in the generalized gradient approximation (GGA), and hybrid functionals: (**b**) HSE, (**c**) B3LYP, and (**d**) PBE0, as well as (**e**) using the method multi-particle perturbations G0W0 using the wave functions of the PBE functional. The maximum valence and minimum conduction bands are shown in bold lines. Indirect transitions determining the band gap Eg are also shown for each case. (Adapted with permission from [7]; AIP Publishing, 2018).

Interestingly, the energy scale of above DOS calculations is directly comparable to the band structure obtained in [7] calculations (see Figure 5a for PBE).

Figure 5a–d compare the electronic band structure obtained using different exchange-correlation functionals (PBE, HSE, B3LYP, and PBE0) with the analogous *G*0*W*0 quasi-particle calculation shown in Figure 5e [7]. The band energies along the several high-symmetry directions were obtained by interpolation of the first-principles data using Wannier90 [65].

The shape of the PBE band structure in Figure 3a is indistinguishable from that reported by Slepko and Demkov [34] displaying a low-dispersive valence band top and a high dispersive conduction band bottom (thick bands). Dispersion of the conduction band minimum states is considerably more pronounced along directions parallel to the *c*-axis (Γ–*A*, *K*–*H**,*** and *M*–*L*), indicating a stronger carrier delocalization and mobility along the main axis. This property could be explored for tuning HAP electrical conductivity through n-type doping or for photo-current measurements. On the other hand, p-type doping is not expected to be beneficial. The valence band top states show very little dispersion, and their heavy holes imply a relatively lower mobility.

Also, in agreement with [34] we find HAP to be an indirect-gap material with *E*g = 5.23 eV at the PBE level. The conduction band minimum is located at **k** = Γ, while the valence band top energy was found somewhere along Γ–*K* or Γ–*M*. The valence band maximum along Γ–*M* is only 0.1 meV higher than the one along ~Γ–*K*. We note that this picture was the same regardless of the functional used, including when using the *G*_0_*W*_0_ method.

The band structure obtained within the HSE06 level is shown in Figure 5b. The increase in the band gap width by more than 30% with respect to the PBE result is self-evident. Using HSE06, we obtain *E*g = 7.11 eV. Often, the band energies are offset in order to lock the valence band top at the origin of the energy scale. We did not follow this procedure and that allowed us to disclose how the gap change depends on the shift of both valence band and conduction band states.

Figure 5b–d show that admixing a fraction of Fock exchange with the semi-local exchange energy has a profound effect on both valence and conduction band states. Consequently, the use of hybrid functionals has implications not only to the accuracy of calculated defect-related or inter-band transitions (e.g., observed in luminescence or UV-VIS absorption), but also to transitions involving core or vacuum states (e.g., observed in electron photoemission or core electron energy loss spectroscopy) [7].

The gap of the B3LYP band structure depicted in Figure 5c is 0.6 eV narrower than that reported by Corno et al. [35] using the same functional. Again, the difference is likely to be due to the unsuitability of the atomic-like basis employed in [35], which resulted in the under-screening of the band structure. The band gap width and the band gap edge energies obtained at the B3LYP-level are closer to the *G*0*W*0 results than any other functional.

Thus, here it should be noted and concluded that with the advent of each of the next developed types of DFT functional, the total calculated electronic band structure of pure HAP is change: the energies of valence band top is down and the bottom of conductive band is up. As result, the width of the forbidden band gap Eg = Ec − Ev increases (see Figure 5a–e). 

This trend of the calculated band gaps agrees with that obtained by Garza and Scuseria [66] for an eclectic mix of semiconductors and insulators. Accordingly, HSE06 and B3LYP showed a closer correlation with the experiments (the former giving slightly smaller gaps overall), whereas inclusion of larger fractions of exact exchange, like in PBE0, led to an overestimation of Eg. This ordering also suggests that the gap width obtained within G_0_W_0_ (Eg = 7.4 eV) should be close to the real figure.

Another interesting point should be noted here, regarding the origin of the bottom of the conduction band, in particular, the highly dispersive bands shown in Figure 5, the situation here is somewhat controversial. In [34], an analysis of LDOS at the bottom of the conduction showed that the lowest energy bands originate from Ca(4s) states.

The authors in [7] argue that this view finds several difficulties. First, in [7], the authors find the onset of the Ca-LDOS (E_onset_ = 8.40 eV) located ~1 eV above the conduction band minimum energy. Within that energy range, all that can be related to Ca are the flat Ca(3d) bands above 8.4 eV. Second, right Figure 4e shows that states just above Ec have a considerable localization on OH molecules.

Interestingly, in this case the highly dispersive bands that form the bottom of the conduction band of HAP are anti-bonding states from an infinite …OH–OH–… hydrogen bridge sequence (along OH-channel through all HAP crystal structure), much like a 1D-ice phase. This statement finds support in the band structure of hexagonal ice (see, e.g., Figure 4 and Figure 6 in [67,68]). From comparison of these band structure images with Figure 5a, it becomes immediately evident that the dispersion shape of the lowest conduction band of HAP is analogous to that of the hexagonal ice.

In this relation, it is interesting to note that nano-confined water [66] inside hydrophilic cavity (or inner channel) inside so-called peptide nanotube (such as, e.g., the diphenylalanine nanotube) has structure close to the one-line hexagonal ice structure [69,70]. So, we should note here that it is a common property in tubular or columnar molecular-like periodical crystal structure, which could be observed in these related structures.

#### 3.2.3. Electronic Band Structure of Defective HAP (with OH-Vacancy and O-Vacancies)

**Figure 6:**
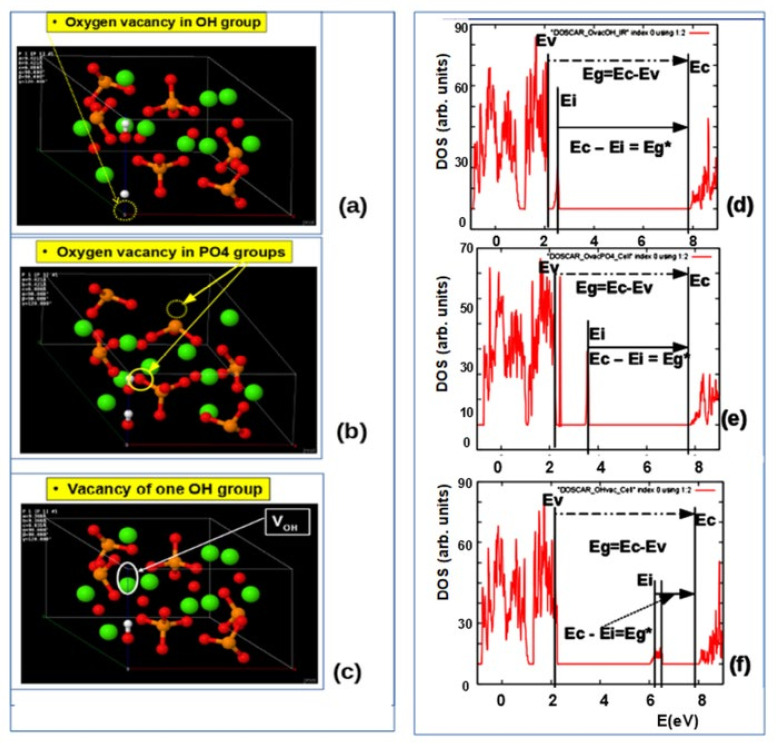
Density of electronic states (DOS) for HAP unit cell with defects: (**a**) HAP unit cell model with O vacancies from the OH group; **(b**) HAP unit cell model with O vacancies from the PO_4_ groups (this may be with O atom from different positions); (**c**) full vacancy of the OH group from HAP unit cell model; (**d**) DOS for case of the O vacancy in OH group; (**e**) DOS for case of the O vacancy in PO_4_ group; (**f**) DOS for case of the full OH vacancy. (Data presented here obtained by calculations firstly using AIMPRO (LDA) [5,6,38] and then VASP (GGA) [39], similar to our works [4,5,6,7,8]). (Adapted with permission from [5]; IOP Publishing, 2015).

In Figure 6, examples of DOS in the results of GGA (PBE) calculations are given for defects in HAP structure, such as O vacancies from the OH group, O vacancies from the PO_4_ group, and a full vacancy of the OH group, based on HAP unit cell model with 44 atoms.

The data of calculated energies of the electronic band structure’s basic states are presented in Table 4.

These data are close to results of other authors [19,30,31,32,33,34]. The results obtained also lead to a small shift in all level energies for defects in the calculations of the GGA (PBE) methods relative to LDA (Table 3). Both methods predict changes in the band gap Eg and the electron work function Δϕ, which can be measured experimentally [2,4,9,53]. To compare this change in the band gap Eg in the presence of a defect in the HAP and to distinguish it from defect-free HAP, we here introduced the designation for the case of a defect-free HAP as Eg0, and then the relative change in the band width will be ΔEg = Eg − Eg0, where Eg is the band gap width in the case of a HAP with a defect. The electron work function ϕ has approximately the same changes Δϕ ≈ ΔEg (it is important for comparison of such changes with experimental data of the photoelectron emissions) [4,5,9]. All these computed changes are summarized in Table 4 below.

In addition, we will denote the energy of photoexcitation of an electron from the local level Ei to the conduction band Ec, which arises in the presence of a defect, as Eg* = Ec − Ei, and this will correspond to the new effective width of the band gap Eg* for optical excitation of an electron and/or photoabsorption of light, which are usually observed in the experiment. These designations are also used in Figure 6 and others figures and tables below.

Similarly, here we introduce the excitation energy of an electron from the valence band Ev to this level Ei, as Ev − Ei, which is presented in Table 4.

It is important to note that the levels of optical absorption for OH vacancies in HAP, established here (Table 3), lie in the region of ~1.6–2.3 eV and they are practically not observed in the experiment, even at a significant concentration of OH vacancies. The calculated density of electronic states (DOS) from the HAP data shows that the intensity of the DOS peaks for the energy levels Ei caused by OH vacancies (located approximately in the middle of the band gap Eg) is very low, compared with the intensity of the main DOS peaks (the top of the valence band Ev and the bottom of the conduction band Ec, which determine the band gap Eg = Ec − Ev) [5]. Therefore, this transition is difficult to detect experimentally by photoelectron excitation. This turns out to be possible only by the photo-luminescence method, upon excitation of a significant number of electrons and from deep valence band levels into the conduction band by the method of synchrotron irradiation [53]. However, in this case, namely the changed value of the optical absorption can be experimentally recorded, which rise up to the value Eg* ~5.3–5.5 eV, due to rise of HAP band gap caused by OH vacancies (Table 3). 

At present, work on the experimental detection and observation of these spectral characteristics of optical absorption in this energy range is underway, created by such defects, as the vacancies of OH groups in HAP, are being actively carried out and are in progress.

**Figure 7:**
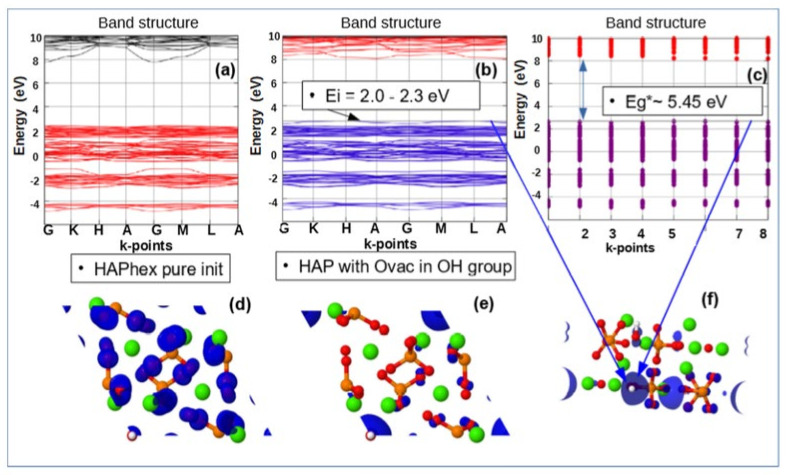
Band structure and partial charge density distribution for O-vacancy from OH group in hexagonal HAP (for one unit cell model): (**a**,**d**) band structure and partial charge density (top view) of initial pure HAP; (**b**,**e**) band structure and partial charge density (top view) for HAP with O-vacancy in OH group (local energy level Ei ~2.0–2.3 eV is close to the top of valence band ΔE = Ei − Ev ~0.1–0.3 eV); (**c**) band structure distribution along k-points with local energy level leaded to optical Eg*~5.45 eV; (**f**) partial charge density for electrons with their eigenvalues around the energy level Ei of the O-vacancy in HAP (side view of the unit cell).

**Figure 8:**
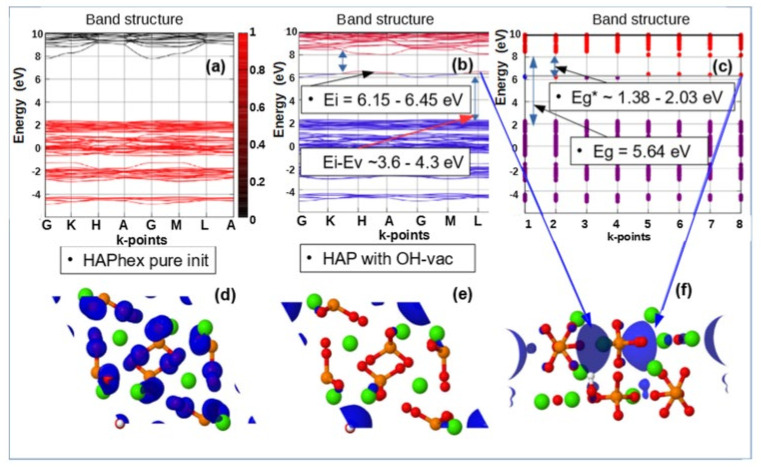
Band structure and partial charge density distribution for OH-vacancy in hexagonal HAP (for one unit cell model): (**a**,**d**) band structure and partial charge density (top view) of initial pure HAP; (**b**,**e**) band structure and partial charge density (top view) for HAP with OH-vacancy (local energy level Ei ~6.15–6.45 eV is the middle of the forbidden band and with ΔE = Ei-Ev~3.6–4.3 eV); (**c**) band structure distribution along k-points with local energy level leaded to optical Eg*~1.38–2.03 eV in comparison with total Eg ~5.64 eV; (**f**) partial charge density for electrons with their eigenvalues around the energy level Ei of the OH-vacancy in HAP (side view of the unit cell).

**Figure 9:**
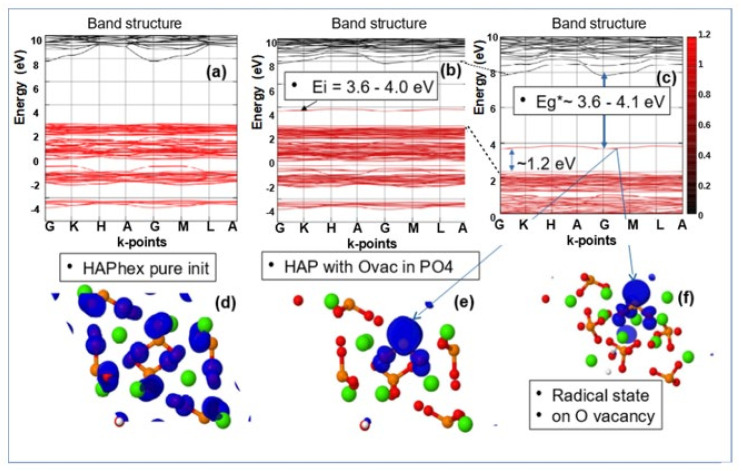
Band structure and partial charge density distribution for O-vacancy from PO_4_ group in hexagonal HAP (for one unit cell model): (**a**,**d**) band structure and partial charge density (top view) of initial pure HAP; (**b**,**e**) band structure and partial charge density (top view) for HAP with O-vacancy from PO4 (local energy level Ei ~3.6–4.0 eV is the middle of the Eg band and with ΔE = Ei − Ev ~1.1–1.3 eV); (**c**) band structure distribution along k-points with local energy level leaded to optical Eg*~3.6–4.1 eV in comparison with total Eg ~5.34 eV; (**f)** partial charge density for electrons with their eigenvalues around the energy level Ei of the O-vacancy of PO_4_ group in HAP (side view of the unit cell).

Figure 7, Figure 8, and Figure 9 below show examples of the band structure and the partial charge density for electrons with their eigenvalues in the range specified around the energy level Ei of corresponding defect structure for main defects in HAP, obtained after optimized VASP calculations in one unit cell model.

These results clearly show that it is precisely the oxygen vacancies of the PO_4_ group that provide the optical band with the energy Eg* = ~3.6–4.2 eV, which is usually observed in many experimentally prepared samples [4,5,6,19,20,21,22,36,37,53]. All these samples in the manufacturing process undergo heat treatment at temperatures T = 700–1200 ° C (that is ~973–1473 K) and this leads to the formation of not only OH vacancies, but also a multitude of O vacancies [19,20,21,22] (especially from the PO_4_ group). This leads to the observed optical absorption and optical band width Eg* ~3.6–4.2 eV, while the band gap of the defect-free perfect stoichiometric HAP turns out to be wider and equal to Eg~5.4 eV and more (in the GGA (PBE) calculations). 

Note that the existing various experimental data [6,9,20] demonstrate optical absorption in HAP samples just in this energy range. In addition, these calculation data correlate with other, more accurate calculations using more advanced methods and models, considered below in the Section 3.3 (Section 3.3.1, Section 3.3.2). This means that even simpler models and methods correctly reflect the essence of changes in the physical properties of HAP in the presence of defects in its structure.

Further development of the calculations was the transition to calculations from one unit cell to a super-cell model consisting from of 2 × 2 × 2 = 8 unit cells, (space group P6_3_, comprising a total of 352 atoms), as well as the application of the hybrid functional PBE (for 1st step optimization) in combination with B3LYP (for2nd step calculation) using DFT method [7,8]. This made a possibility to classify different types of oxygen vacancies of the PO_4_ group more correctly and accurately, which increases the calculation accuracy and to *highlight not only neutral defects, but new more complex types of defects*—*extended charged complex oxygen vacancies.*

These new structural extended (or bridging structures) defects can play an important role in changing the properties of electrically charged HAP.

### 3.3. Electronic and Optical Properties of HAP with Defects in Supercell Model

#### 3.3.1. Oxygen Originated Complex HAP Defects in Supercell Model

**Figure 10:**
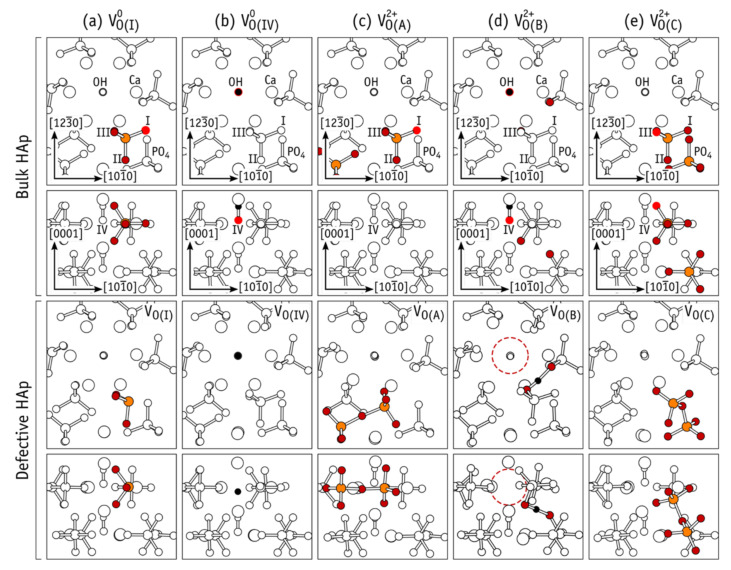
Diagrams showing “Bulk” (upper half) and “Defective” (lower half) HAP. For each row pair, upper and lower rows display the same structures viewed along the [0001] and [1230] directions of the hexagonal lattice, respectively. Formation of structures I, IV, A, B, and C of V_O_ defects is explained in columns (**a**–**e**), respectively. Only atoms belonging to the core of the defect are colored (P, O, and H atoms are shown in orange, red, and black, respectively). Vacancies were created by removing the bright red O atom shown in the “Bulk” figures. Upon atomic relaxation, the resulting structures are those in the corresponding “Defective” figures. Reprinted with permission from [8]. (Adapted with permission from [8]. Copyright (2019) American Chemical Society).

Performing these series of calculations, it was found that different types of defects arise, if the symmetry of the atomic group associated with different spatial arrangement of various atoms in the PO_4_ group. Besides, usual oxygen vacancy V_O_, here arises complex extended defects, which depend on their charge state Q = 0, +1, +2. (Figure 10) [8,36,37]. The O vacancy of OH group influences the formation of new complex defects because of charge Q variation.

Crystalline HAP has oxygen atoms on four symmetries in equivalent sites, which are referred to as oxygen types I−IV. Types I, II, and III in the phosphate units, while type IV oxygen atoms are located in the OH^-^ anions. These are denoted as O(I), …, O(IV) and are shown in the upper half of Figure 10, where portions of bulk HAP are depicted. We note that PO_4_ groups have two nearly symmetric O(III) atoms which are superimposed in the upper view of bulk HAP in Figure 10. 

Among the many vacancy structures investigated, those shown in Figure 10a−e are the most relevant as they showed lower energy. Metastable structures with more than 2 eV above the ground state (for each particular charge state) will not be discussed. Figure 10a describes the formation of a pyramidal PO_3_ structure, where removal of the O(I) atom in “Bulk HAP” highlighted using a bright red color, leads to V_O(I)_ as shown in the lower part of the figure, where a “Defective HAP” region is shown. 

**Figure 11:**
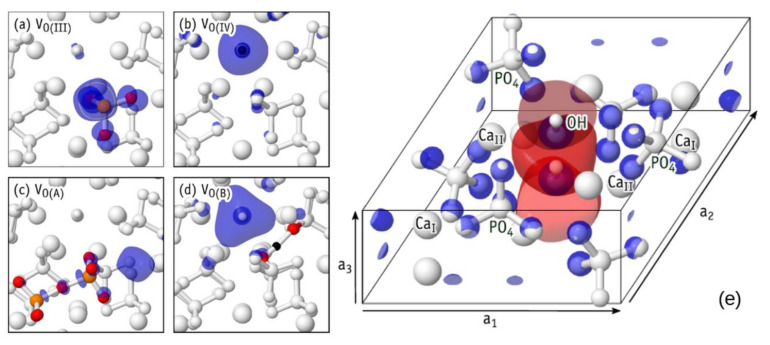
Electron density isosurfaces from the highest occupied Kohn−Sham level of neutral V_O_ defects in HAP. The density of V_O(III)_ in (**a**) is representative of V_O(I)_ and V_O(II)_ as well. The density of V_O(IV)_ in (**b**) shows the case of a missing O(IV) atom, leaving an isolated H atom in the OH channel. The density of extended charged defects V_O(A)_ and V_O(B)_ are shown in (**c**,**d**) (description is in text). Isosurfaces are drawn at constant electron density n = 0.001 Å^−3^. Reprinted with permission from [8]. Copyright (2019) American Chemical Society. (**e**) Lowest unoccupied Kohn-Sham state (bottom of the conduction band) of a HAP at **k** = G. Blue and red isosurfaces represent y(**r**) = +0.02 and y(**r**) = 0.02 phases of the wave function, respectively. All atoms are shown in white. Reprinted with permission from [7]. (Adapted with permission from [7]. CCC (2018) AIP Publishing).

Analogous structures for V_O(II)_ and V_O(III)_ were obtained as well. In the neutral charge state, the resulting [PO_3_^3−^]_PO4_^0^ structures display a fully occupied sp^3^ orbital on the P atom, resembling the phosphine molecule. This is shown in Figure 11a, where an isosurface of the electron density corresponding to the highest occupied state is represented in blue for the specific case of V_O(III)_^0^.

Figure 11b shows the case of a missing O(IV) atom, leaving an isolated H atom in the OH channel. This defect can be rationalized as the removal of neutral O from OH^−^, leaving a hydride anion, and maintaining charge neutrality of the whole system. In the neutral charge state, we have a [H^−^]_OH_^0^ structure, where after structural relaxation, the hydride species becomes located close to the site of the missing O(IV) atom (compare lower diagrams of bulk and defective HAP in Figure 10b).

The density corresponding to the highest occupied state of V_O(IV)_^0^ is represented in Figure 11b, which clearly shows the formation of a hydride anion in the OH channel. Figure 11e shows the OH-vacancy defect [7]. In V_O(I)_−V_O(IV)_ defects, all atoms (except the missing oxygen) remain essentially close to their original crystalline coordinates, hence the use of subscripted O(I)−O(IV) labels to identify their structure. However, additional structures, hereafter referred to as ***extended structures***, were also found for the oxygen vacancy in HAP [8]. One type of such extended structures can be described as a pair of neighboring oxygen vacancies connected by an O-interstitial, 2V_O_ + O. Another 2nd type is best described as a complex made of an OH-vacancy next to an H-interstitial, V_OH_ + H. Two defects of type 2V_O_ + O are singled out and labeled V_O(A)_ and V_O(C)_. They are shown in Figure 10c,e, respectively. One V_OH_ + H defect is shown in Figure 10d and is referred to as V_O(B)_. These new defect structures are very important for practices too.

A dashed circle is used in the figure to highlight the missing OH unit. The highest occupied state of the extended structures is shown in Figure 11c,d. They either overlap the void regions of the HAP crystal or the vacant volume of the OH-channel, thus suggesting that they are donors with antibonding character or strong resonance with conduction band states [7].

For relaxations in charge state +2 that started from structures I and III, the final structures were, respectively, A and C. Here, the P atom of the PO_3_ unit in V_O(I)_ (or V_O(III)_) moved across the plane defined by the three O atoms to connect to the O atom from the nearest PO_4_ moiety. Such a severe relaxation can be explained by electron transfer from a neighboring PO_4_^3−^ anion to the empty P(sp^3^) orbital of [PO_3_^−^]_PO4_^2+^ in V_O(I)_^2+^ or V_O(III)_^2+^ (see Figure 11a) and the subsequent formation a new P−O bond. The result is an extended [PO_3_^2−^−O−PO_3_^2−^]_2(PO4)_^2+^ structure shown in Figure 10c,e.

When initiating the relaxation in charge state +2 from structures II and IV, the resulting configuration was in both cases V_O(B)_^2+^. In this charge state, the defect comprises an interstitial H+ next to a positively charged OH vacancy, that is, [PO_4_^3−^−H^+^−PO_4_^3−^]_2PO4_^+^ + V_OH_^+^. The proton is located on a high electron density site between two oxygen anions. The net positive charge of the OH vacancy follows from depletion of two electrons from the channel-state represented by the isosurface of Figure 11d. When starting from structure II, Coulomb attraction and subsequent reaction between neighboring OH^−^ and [PO_3_^−^]_PO4_^2+^ leads to the formation of the V_O(B)_^2+^ extended structure. Alternatively, when starting from structure IV, a proton in the initial [H^+^]_OH_^2+^ configuration is strongly attracted by O-anions in neighboring PO_4_^3−^ moieties, also ending up in V_O(B)_^2+^ as depicted in Figure 10d.

The study of transitions between the energy levels of such more complex defects (including at different levels of the charge state of the HAP crystal, and different types of its conductivity: electron n-type, or hole p-type) showed that the transition of electrons can also include a phononless transition with energy ~3.6–3.9 eV [8,36,37]. This mechanism can also explain the onset of absorption at 3.4–4.0 eV in the experimental observation of photocatalysis in HAP under constant UV illumination [6,8].

The results obtained convincingly show the primary role of defects such as oxygen vacancies and vacancy of OH group in the formation of actually observed values in the range approximately of ~3.6–4.3 eV for oxygen vacancies Ovac {VO(I), VO(II), VO(III)} from PO_4_ groups, and at ~5.06 eV for oxygen vacancy VO(IV) from OH group (see below in Section 3.3.3) for the effective optical absorption and excitation bands Eg* in various samples of HAP materials [5,6,7,8,9]. At the same time, for example, the main experimentally observed values of the optical absorption band according to data measured UV spectra for the HAP samples of marine origin, and the commercial ones, respectively, (presented in Table 2 and Table 4 in work [6]) lie in the close range approximately of ~3.4–4.2 eV (which could be arisen by oxygen vacancy from PO_4_ groups) and ~5.05 eV (which could be arisen by oxygen vacancy from OH group). These data directly correspond to the data calculated in our computational studies presented and discussed in this article.

Nevertheless, the issues related to obtaining more accurate, fair, and correct numerical results, which are very important for many practical applications, as well as the issues of statistics (and dynamics) of accumulation and annealing of defects in various unit cells in the entire volume of the crystal, remain rather complex problems and require further research.

In addition, it is obvious that all these points require additional experimental observations and research. This work must be carried out in a complex.

These works are now in progress in the computational direction, and new results will soon be obtained in this direction of calculating defects in HAP, including, in addition to the types of vacancies here considered, also other defects of the type of insertions and substitutions of some atoms in HAP unit cell lattice.

#### 3.3.2. Kohn−Sham Energy Levels of Neutral Oxygen Vacancy V_O_ Defects 

Inspection of the Kohn−Sham eigenvalues at **k** = Γ confirmed that neutral vacancies V_O_ defects are all donors [8,37]. The calculations performed in these cases, taking into account the even more accurate B3LYP calculation scheme, showed similar shifts in the energy levels created by defects such as oxygen vacancies of the PO_4_ group, and their main contribution, which determines the change in optical properties and the change in the work function, remains at a level of ~ 1 eV or less (Table 5), despite the differences in the calculations of the band gap Eg for initially perfect stoichiometric defect-free HAP.

**Figure 12:**
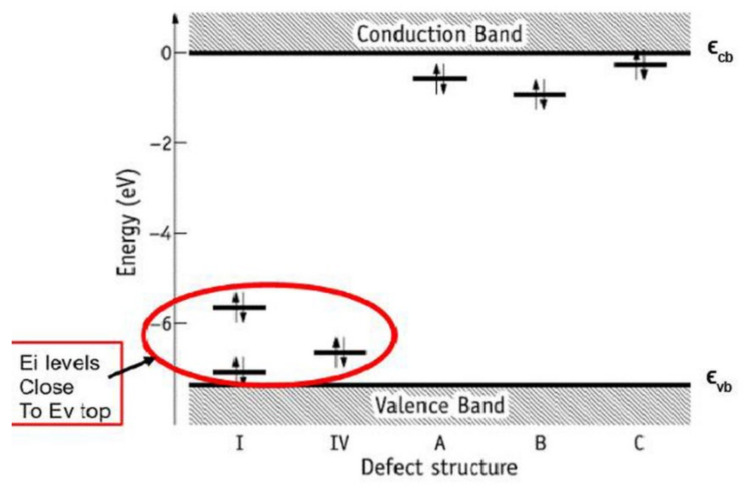
Kohn−Sham energy levels of neutral V_O_ defects in a HAP supercell at the **k** = Γ point. The defect structure I is also representative of structures II and III (see text). The latter have gap states that deviate from those of V_O(I)_ by less than 0.2 eV [33]. Adapted with permission from [8]. (Copyright (2019) American Chemical Society).

As can be seen, regardless of the method of calculation, oxygen vacancies of the OH and PO_4_ groups (in the absence of charge in these defects, i.e., at Q = 0) form a group of energy levels (Figure 12) located close to the top of the valence band and they are donor electrons [8,48] (electron acceptors, that is, levels with a negative charge were not found).

In this case, the shift of the levels of oxygen vacancies in the PO_4_ group is ~1.15–1.65 eV upward from Ev, and for a vacancy from the OH group, ~0.4–0.7 eV, and this also corresponds to a change in the electron work function during the formation of such defects. As everyone can see, the positions of these energy levels are not very different (especially in the case of PBE calculations), although in the case of B3LYP these levels are slightly higher than Ev in the direct case. In any case, these deviations are within 1 eV.

It is also important to note that such levels close at the top of the valence band have recently been observed in experiments on photoelectron emission spectroscopy, and the work function of a photoelectron from HAP was measured for various external influences [2,4,5,53]. In addition, in [53] it was noted that such energy levels (and photoelectrons emitted from these energy levels) can arise under a number of actions on HAP samples (heating and annealing, gamma irradiation, microwave effects, and combined hydrogenation with microwave radiation).

In these cases, a sufficiently large number of oxygen vacancies (also in the OH group) can be induced having the lowest energies levels measured from the top of the valence band (see Table 4 and Figure 12).

#### 3.3.3. OH-Vacancy in HAP Supercell Model and Some General Remarks

The vacancy of one OH group in the HAP supercell model was considered in a similar way. In this case, calculation with the GGA approximation in the hybrid functional PBE and B3LYP was performed using VASP [39] and Quantum ESPRESSO [41].

**Figure 13:**
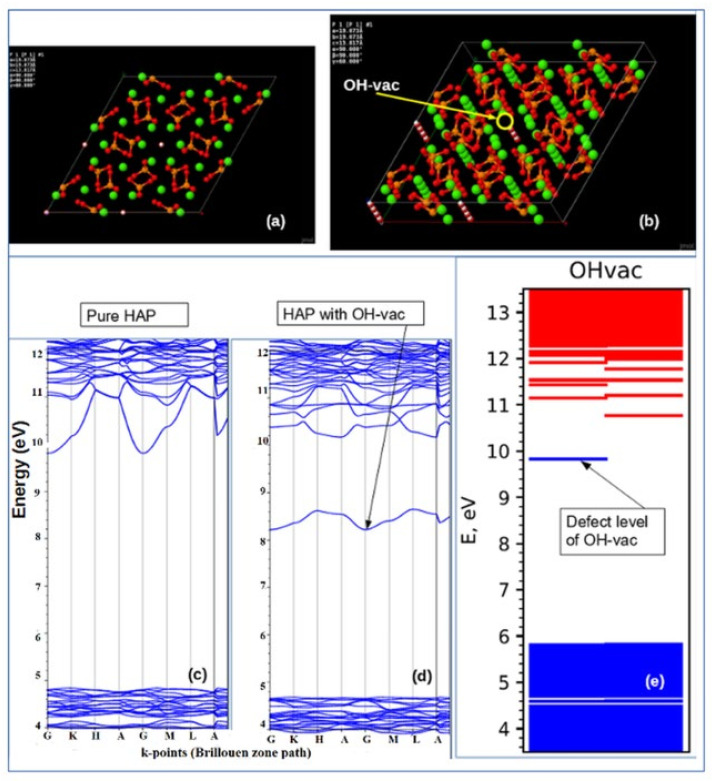
Model of OH-vacancy in HAP supercell: (**a**) top view on HAP supercell; (**b**) OH-vacancy in HAP supercell (iso projection); (**c**) band structure of pure HAP; (**d**) band structure of HAP with OH-vacancy computed using VASP; (**e**) band structure of HAP with defect level from OH-vacancy computed using Quantum ESPRESSO.

Figure 13 shows the results obtained in this case. After PBE optimization and B3LYP calculation using VASP energy band structure for HAP with OH-vacancy (Figure 13b), the following data are presented: Ev = 0.726 eV, Ec = 8.066 eV and Eg = Ec − Ev = 8.066 − 0.726 = 7.34 eV; the defective energy level due to OH-vacancy Ei = 5.3586 eV (occupied, spin up) and resulted optical gap from this energy level is equal Eg* = Ec − Ei = 8.066 − 5.3586 = 2.7074 eV. This data is in line with previous data obtained using another similar method in one unit cell model.

In addition, it is important to note that the energy of formation of defects in the HAP crystal [7,8] was considered and investigated also in the formalism with a chemical potential based on work [52], which describes the procedure for calculating the energies of formation of defects in a similar study. This formalism also takes into account the formation of defects with an excess of electrons Δ*n_e_* (with respect to the neutral state) when they are capable of capturing/emitting electrons from/to an electronic reservoir with a chemical potential *μ_ε_.* The results obtained in this approach allow more correct calculations taking into account the thermodynamics of the formation of a defect with a screened charge as ***a quasi-particle*** in a crystal [7,8]. As a result, we obtain corrections to the energies, including the energies of optical properties and transitions, which, in our opinion, turn out to be even closer to those observed experimentally. These generalized data are shown here in Table 6.

The results obtained convincingly show the primary role of defects such as oxygen vacancies and vacancy of OH group in the formation of actually observed values of the effective optical absorption and excitation bands Eg* in various samples of HAP materials. These works are in progress and new results will soon be obtained in this direction of calculating defects in HAP, including, in addition to the types of vacancies here considered, also other defects of the type of insertions and substitutions of some atoms in HAP unit cell lattice.

## 4. Conclusions

Computer studies of defects in hydroxyapatite, carried out by various methods, show their significant influence both on the structural and mechanical, as well as on the electronic and optical properties of hydroxyapatite with such defects. This is very important for practical applications. In this review, we consider in more detail defects such as OH group vacancies and oxygen vacancies from different PO4 groups and OH groups as well. Calculations performed by different methods showed that these vacancies significantly change, first of all, the electronic and optical properties of HAP. In this case, the vacancy of the entire OH group leads to an entire absorption band in the range ~1.4–2.4 eV (with several DOS peaks). It is close to the red color of optical spectrum. However, the intensity of these DOS is small compared to the DOS of the top of the valence band. As a result, it is practically impossible to register this range of optical absorption (it is at the level of measurement errors). At the same time, an OH vacancy leads to a noticeable increase in the band gap of the order of ΔEg ~0.5–0.9 eV. This leads to a change in the optical absorption and also a change in the work function of the electron exit, which is recorded in the experiment. For example, it can be the level Eg ~5.5–5.75 eV, which belongs to the ultraviolet spectrum and is close to the observed optical absorption of HAP samples with OH vacancies (created in them experimentally upon heating and subsequent cooling).

Oxygen vacancies have very different optical properties. Oxygen vacancies arising from various oxygen atoms of the PO_4_ group and the OH group turn out to be of different types and depend on the symmetry of the corresponding group. As it was shown in [7] that besides neutral O-vacancy here may exist charged oxygen vacancies also form not only point defects, but more complex defects—extended or bridging (in the case of a charge Q = +2, Figure 10). The latter reconstructing to point-like defects (at Q = +1 and = 0). This rearrangement from extended to point defects occurs with bond breaking and causes also optical absorption effects. This transition leads to spontaneous rupture of bridging P-O-P or O-H-O bonds at extended defects and, most likely, explains the onset of absorption at 3.4–4.0 eV for observing photocatalysis under constant ultraviolet illumination [6]. It is important that these types of structure and the stability of defects strongly depend on the charge states.

It was found that the oxygen vacancies essentially occur in such two distinct forms, either as a simple vacant oxygen site (referred to as structures I-IV, Figure 10a), or as an oxygen atom replacing two neighboring oxygen vacancies (bridge or extended structures named as “A–C”, Figure 10c–e). The former type of vacancies is deep donors, while the latter are shallow donors with rather low ionization energies. No acceptor states (stable negatively charged defects) were found. Vacancy structures I-IV are more stable in the neutral charge state, while bridge structures A-C are preferred in the double plus charge state. This means that the oxygen vacancy adopts rather different configurations on samples where the Fermi energy is in the upper or the lower half of the band gap. As regards to the neutral O-vacancy, corresponding inspection of the Kohn-Sham eigenvalues at k = Γ confirmed that neutral oxygen vacancy defects are all donors involving the luminescence and absorption of ~3.6–4.2 eV.

Further development and more accurate calculation of these electronic properties and optical photoexciting and photocatalytic processes can be made also by addition-correct calculations of the electron–electron correlation of the excited electron states taking into account the Frank Condon (FC) relaxation. In [36,37], these contributions are calculated for the case of the V_O_(IV) vacancy in HAP P6_3_ supercell model and their results showed that the FC shift obtained has reasonable values. These investigations should be continued for all other oxygen vacancy types.

Despite the need to continue further comprehensive study of HAP defects, however, based on the results already obtained so far, we must conclude that for any case, irrespective of these FC relaxation processes, the formation of the various types defects in HAP through different oxygen vacancies gives rise to an opportunity of the photo-excitation processes in the close ultraviolet (UV-C) and in the visible light region. These photo-excitation effects obviously produce the photo-catalytic activity of HAP, as well as provide the changes in the photoelectron work function and surface electric potential, which is important for implant covered by HAP. One only needs to introduce a sufficient amount of these oxygen vacancies by some external actions, such as heating/annealing, gamma-irradiation, and combined hydrogenation with microwave irradiations [71]. The electron photoexcitation from additional energy levels (with Eg* = ~3.4–4.2 eV), which arose due to oxygen vacancies inside the forbidden band gap, can provide this electronic and optical properties of the treated HAP samples.

## Figures and Tables

**Table 1:** Unit cell parameters *a,b,c* [Å] (from [11]).

Phase	Group	*a,* Å	*b,* Å	*c,* Å
Hexagonal	P6_3_/m	9.417	9.417	6.875
Monoclinic	P2_1_/b	9.480	18.960	6.830

**Table 2:** Data of HAP lattice parameters and bulk modulus for pristine HAP and HAP with OH-vacancy.

Property	Experim. [11]	Experim. [58]	Experim. [59] ([60])	AIMPRO (LDA) ^(3)^ [5,6]		PBE VASP ^(3)^ (GGA)		PBE (GGA)/SuperCell ^(4)^ [7,8]	B3LYP/Super-Cell ^(4)^ [7,8]	HSE [7]	PBE0 [7]
				OH-OH P6_3_	OH-HO P6_3_/m	OH-OH P6_3_	OH-HO P6_3_/m				
Initial stoichiometric HAP (hexagonal P6_3_)											
*a*, Å	9.417	9.4236	9.4205 (9.4248)	9.4732	9.4624	9.3628	9.3640	9.537	9.5770	9.481	9.477
*c*, Å	6.875	6.8802	6.8828 (6.8860)	6.9986	7.0182	6.8454	6.8621	6.909	6.8767	6.859	6.851
V, Å ^(3)^	527.99	529.13	528.99 (529.71)	543.92	544.20	519.69	521.09	546.07	546.22	533.95	532.88
B,GPa	89 ± 1 ^(1)^			81.6 ± 2	82.35 ± 2	-		82 ± 3	86 ± 2	83 ± 3	82.8 ± 0.3
E, a.u. E, eV				−467.0992	−467.0944	−311.82	−311.39				
ΔE,eV				0.132		0.43					
HAP with OH-vacancy											
*a*, Å		9.4155 ^(2)^		9.4883		9.3685	9.4210	9.537	9.5770	-	-
*c*, Å		6.8835 ^(2)^		7.0018		6.8351	6.8800	6.909	6.8767	-	-
V, Å ^(3)^		528.48 ^(2)^		545.905		519.53	528.83	546.07	546.22	-	-
B,GPa				78 ± 2		−297.78	−297.99				
E, eV						0.21					

^(1)^ References [61,62]. ^(2)^ Data from Bulina N.V. experiment (cooling from 1100 °C in He atm.—comment is in the Section 3.1.1 below). ^(3)^ OH-OH is the ordered P6_3_ phase and OH-HO is the disordered P6_3_/m phase—comment 1 in the text. ^(4)^ Two-steps calculation for supercell models—comment 2 in the text.

**Table 3:** The calculated HAP lattice parameters with various type of the O-vacancy.

Property	Type of O-Vacancy	AIMPRO (LDA) [5,6]	PBE–VASP (GGA)
HAP with O-vacancy from OH (one unit cell HAP P6_3_ model)			
*a*, Å	-	9.4539	9.3437
*c*, Å	-	7.0028	6.8463
V, Å ^(2)^	-	542.03	517.64
HAP with O-vacancy from PO_4_ (one unit cell model HAP P6_3_ model)			
	Atom O in different positions in PO_4_^(1)^		
*a*, Å,	V_O1 (O6) V_O2 (O15) V_O3 (O30,O35) V_O4 (O24)	9.4599 9.4630 9.4581 9.47295	9.3570 9.3520 9.3544
aver. *a*, Å		9.4635 ± 0.005	9.3545 ± 0.005
*c*, Å	V_O1 (O6) V_O2 (O15) V_O3 (O30,O35) V_O4 (O24)	6.9884 6.9890 6.9893 6.97822	6.8139 6.8242 6.8402
aver. *c*, Å		6.9890 ± 0.005	6.8261 ± 0.005
V, Å ^(2)^	V_O1 (O6) V_O2 (O15) V_O3 (O30,O35) V_O4 (O24)	541.60 542.01 541.47 542.31	517.86 518.33 517.45
aver. V, Å ^(2)^		541.85 ± 0.3	517.88 ± 0.1

^(1)^ O-vacancy from PO_4_: Atom O—is in various positions of PO_4_ (and from different PO4 groups, according with Figure 2). ^(2)^ OH-OH is the ordered P6_3_ phase and OH-HO is the disordered P6_3_/m phase—comment 1 in the text.

**Table 4:** Calculated data for HAP defects by two various approaches (aver. error = ±0.05, ±0.1eV) [4,5,6].

Defect Type	LDA				GGA (PBE)			
	Eg = Ec − Ev, eV	ΔEg = Eg − Eg0 ~ Δϕ, eV	Ei − Ev, eV	Eg* = Ec − Ei, eV	Eg = Ec − Ev, eV	ΔEg = Eg−Eg0~ ~ Δϕ eV	Ei − Ev, eV	Eg* = Ec − Ei, eV
HAP in P6_3_/m, Eg0	4.6	-	-	-	5.26	-	-	-
O_(OH)_ vac	5.15	+0.55	0.1 (1 occ.)	5.05	5.72	+0.46	0.27	5.45
OH vac	5.49	+0.89	3.11–3.82 peaks: 3.40 3.53 3.66 (½ occ.)	2.38–1.67 peaks: 2.09 1.96 1.83	5.75	+0.49	3.66–4.28 peaks: 3.96 4.11 4.17	1.97–1.35 peaks: 1.78 1.63 1.57
Ovac(PO4) ^(1)^ V_O1 (O6) V_O2 (O15) V_O3 (O30,O35) V_O4 (O24)	4.734 4.768 4.735 4.5614		1.346 1.300 1.347 0.9557	3.388 3.468 3.388 3.6057	5.416 5.246 5.326		1.045 1.212 1.142	4.115 4.034 4.184
aver. O_(PO4)_ -vac	4.70 ± 0.2	+0.15	1.14 ± 0.3	3.52 ± 0.3	5.34 ± 0.2	+0.08	1.13 ± 0.2	4.11 ± 0.2

^(1)^ OvacPO_4_: atom O in various positions of PO_4_ and from the different PO_4_ group, according with Figure 2.

**Table 5:** Data for O vacancy from OH and PO_4_ in different position and symmetry (errors ±0.05 eV).

Defect Type	PBE				B3LYP			
	Eg = Ec − Ev, eV	ΔEg = Eg − Eg0 ~Δφ, eV	Ei − Ev, eV	Ec − Ei = Eg*, eV	Eg = Ec − Ev, eV	ΔEg = Eg − Eg0 ~Δφ, eV	Ei − Ev, eV	Ec − Ei = Eg*, eV
HAP in P6_3_/m, Eg0	5.23	-	-	-	7.3	-	-	-
A0=A^0^_I_ (V_O_(I))	5.0674	−0.1626	1.1496 ~1.15	3.9178	7.0497	−0.2503	1.4291 ~1.43	5.6206
A0=A^0^_II_ (V_O_(II))	5.2004	−0.0296	1.3167 ~1.32	3.8837	7.2311	−0.0689	1.6512 ~1.65	5.5799
A0=A^0^_III_ (V_O_(III))	5.1393	−0.0907	1.3811 ~1.38	3.7582	7.1333	−0.1667	1.685 ~1.68	5.4488
D0=D^0^_I_ (V_O_(IV))	5.3004	+0.0704	0.4189 ~0.42	4.8815	7.3842	+0.0842	0.7347 ~0.73	6.6495

**Table 6:** HAP electronic properties with various vacancies Ovac {V_O(I)_, V_O(II)_, V_O(III)_ from PO_4_, V_O(IV)_ from OH} as well as V_OH_ vacancy of OH group—in supercell model; for one unit cell model vacancies Ovac notation are the same as in Table 3: V_O1 (O6), V_O2 (O15), V_O3 (O30,O35), V_O4 (O24) (all energy in eV).

Unit Cell	AIMPRO (LDA), Eg*	VASP-PBE (GGA), Eg*	Super-Cell	PBE opt (Eg* = Ec − Ei) (GGA-Supercell)		B3LYP opt (Eg* = Ec − Ei) (GGA-Supercell)		Spectr.
Ovac from Figure 2			Ovac from Figure 10	Kohn− Sham	Defect as quasiparticle in crystal	Kohn− Sham	Defect as quasiparticle in crystal	
V_O1	3.3880 (Eg = 4.734)	4.115 (Eg = 5.416)	V_O(I)_	3.9178 (Eg = 5.067)	3.7052	5.6206 (Eg = 7.05)	4.3375	UVA–UVB
V_O2	3.6057 (Eg = 4.562)	4.034 (Eg = 5.246)	V_O(II)_	3.8837 (Eg = 5.200)	3.6575	5.5799 (Eg = 7.23)	4.3486	
V_O3	3.4677 (Eg = 4.768)	4.184 (Eg = 5.326)	V_O(III)_	3.7582 (Eg = 5.139)	3.5166	5.4488 (Eg = 7.13)	4.1291	
V_O4	3.6057 (Eg = 4.562)	-	-	-	-	-	-	
Aver. (O1_4)	3.52 ± 0.3 (Eg = 4.70)	4.11 ± 0.3 (Eg = 5.34)	Aver. (I-III)	3.8532 ± 0.2 (Eg = 5.14)	3.6264 ± 0.2	5.5498 ± 0.2 (Eg = 7.14)	4.2717 ± 0.2	UVA
V_O of OH	5.05 (Eg = 5.15)	5.45 (Eg = 5.72)	V_O(IV)_	4.8815	5.0630	6.6495	5.8563	UVC
V_OH_	2.38-1.67 2.09 1.96 1.83 (Eg = 5.49)	1.97- 1.35 1.78 1.63 1.57 (Eg = 5.75)	V_OH_	1.73	1.7372	2.9174 and 2.7074	1.7491 and 2.200	Green-Red
Eg0	4.6	5.26		5.4	5.237	7.34	6.849	

## Data Availability

The data presented in this study are available on request from the corresponding author.
